# Development of an Effective Monoclonal Antibody against Heroin and Its Metabolites Reveals Therapies Have Mistargeted 6-Monoacetylmorphine and Morphine over Heroin

**DOI:** 10.1021/acscentsci.2c00977

**Published:** 2022-10-06

**Authors:** Jinny Claire Lee, Lisa M. Eubanks, Bin Zhou, Kim D. Janda

**Affiliations:** Departments of Chemistry and Immunology, The Skaggs Institute for Chemical Biology, Worm Institute for Research and Medicine (WIRM), The Scripps Research Institute, 10550 North Torrey Pines Road, La Jolla, California 92037, United States

## Abstract

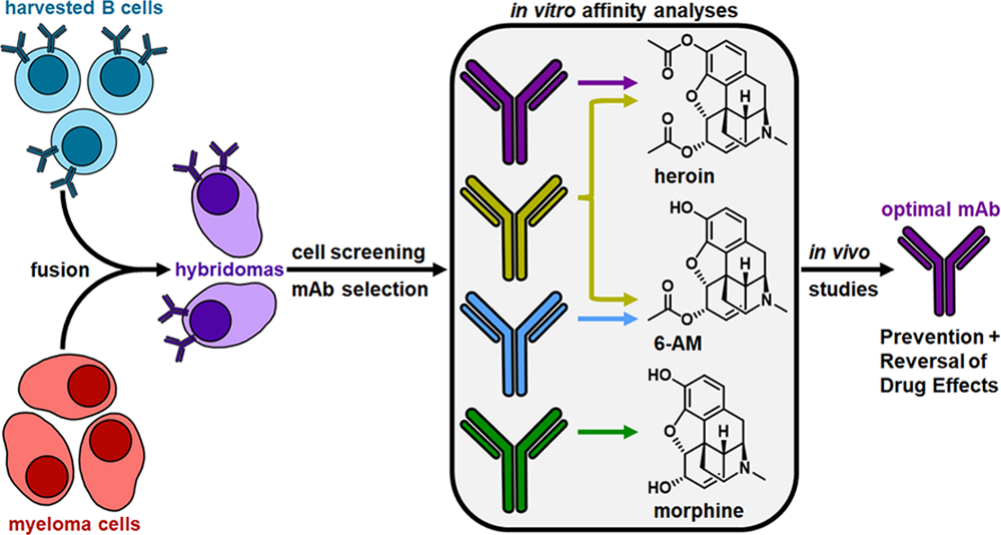

The opioid epidemic is a global public health crisis that has failed to abate with current pharmaceutical treatments. Moreover, these FDA-approved drugs possess numerous problems such as adverse side effects, short half-lives, abuse potential, and recidivism after discontinued use. An alternative treatment model for opioid use disorders is immunopharmacotherapy, where antibodies are produced to inhibit illicit substances by sequestering the drug in the periphery. Immunopharmacotherapeutics against heroin have engaged both active and passive vaccines targeting heroin’s metabolites, 6-monoacetylmorphine (6-AM) and morphine, since decades of research have stated that heroin’s psychoactive and lethal effects are mainly attributed to these compounds. However, concerted efforts to develop effective immunopharmacotherapies against heroin abuse have faced little clinical advancement, suggesting a need for reassessing drug target selection. To address this issue, four unique monoclonal antibodies were procured with distinct affinity to either heroin, 6-AM, or morphine. Examination of these antibodies through *in vitro* and *in vivo* tests revealed monoclonal antibody 11D12 as the optimal therapeutic and provided crucial insights into the key chemical species to target for blunting heroin’s psychoactive and lethal effects. These findings offer clarification into the problematic attempts of therapeutics targeting heroin’s metabolites and provide a path forward for future heroin immunopharmacotherapy development.

## Abstract 2

A monoclonal antibody capable of protecting against heroin’s psychoactive and lethal effects shows decades of research have been mistargeting heroin’s metabolites, 6-monoacetylmorphine and morphine.

## Introduction

For decades, leaders around the globe have tried to address the escalating opioid epidemic. However, since the start of the COVID-19 pandemic, opioid abuse and overdose cases have reached record highs and traditional therapeutic models have not abated the exponential growth of opioid use disorders (OUDs).^1−4^ Treatment for substance use disorders consists of a psychosocial and pharmacotherapeutic compartment,^5,6^ the latter of which has several FDA-approved drugs for opioid abuse—methadone, buprenorphine, naltrexone, and naloxone. These small molecules are problematic due to adverse side-effects, short half-lives, limited availability, high cost, medication adherence complications, therapeutic abuse potential, and relapse to addiction after discontinued use.^7−9^

Due to these disadvantages, another mode of therapy that has been examined to address OUDs is immunopharmacotherapy. This method requires the development of a small-molecule that mimics the drug of abuse called a hapten.^10,11^ The hapten is conjugated to a larger immunogenic protein to aid in immune system recognition of the small hapten antigen.^12,13^ When the hapten-protein conjugate is administered to generate therapeutic polyclonal antibodies against the abused drug, this is considered active immunization. A complementary immunopharmacotherapeutic method is passive immunization, where monoclonal antibodies (mAbs) are obtained and injected into the system to protect against drug effects. Unlike active immunization, passive vaccination does not require lengthy inoculation schedules and provides immediate protection. The main merit of immunopharmacotherapy is that antibodies remain in the periphery while sequestering the drug, thus avoiding the adverse side effects and potential for abuse that traditional pharmaceutics possess.^7,14^ Moreover, this therapeutic model can provide long-term protection, making it more cost-effective and accessible.

Researchers have developed immunopharmacotherapies to address several substance use disorders including cocaine, nicotine, methamphetamine, fentanyl, and hydro/oxycodone.^13,15−17^ Here, hapten design has been a fundamental component for vaccine advancement, and as such, most haptens have been planned to preserve the parent drug’s structure. In contrast, active and passive vaccine development against heroin has been a more daunting task as this illicit opioid is metabolized into multiple psychoactive structures. With these challenges in mind, immunopharmacotherapy endeavors for heroin have focused on a hapten model wherein broad-spectrum protection has been sought against heroin’s metabolites, 6-monoacetylmorphine (6-AM) and morphine,^18−26^ which are produced from heroin through sequential deacetylation by esterase enzymes.^27,28^ This established vaccine paradigm can be attributed to the fact that heroin shows a lower affinity for the μ-opioid-receptor (MOR) than its metabolites,^27,29^ and despite the advancements of these preclinical vaccine reports, there is growing evidence that clinical success is far from certain.^30^

Considering the futile history of heroin vaccine research, we felt to develop an effective immunopharmacotherapeutic against heroin, a departure was needed from previous efforts. Our process led us to consider obtaining mAbs with unique binding characteristics to heroin and its metabolites. Inferences of such an approach would allow us to dissect which of these entities is the crucial chemical species to target with immunopharmacotherapy. Toward this end, four mAbs were generated using a unique deutero-heroin hapten that has been shown to have broad-spectrum affinity to heroin and its metabolites.^22^ The binding profiles of these four mAbs were analyzed using surface plasmon resonance (SPR), which revealed each mAb had nanomolar affinities and unique specificities to either heroin, 6-AM, morphine, or both heroin and 6-AM. Using this data as a foundation, antinociception, pharmacokinetic, and overdose assays were conducted in order to resolve the key chemical species to be targeted for effective blunting of behavioral, toxic, and lethal effects of heroin, which in turn would help reconfigure future heroin immunopharmacotherapy development.

## Results and Discussion

### Monoclonal Antibody Development and Selection

**Figure 1:**
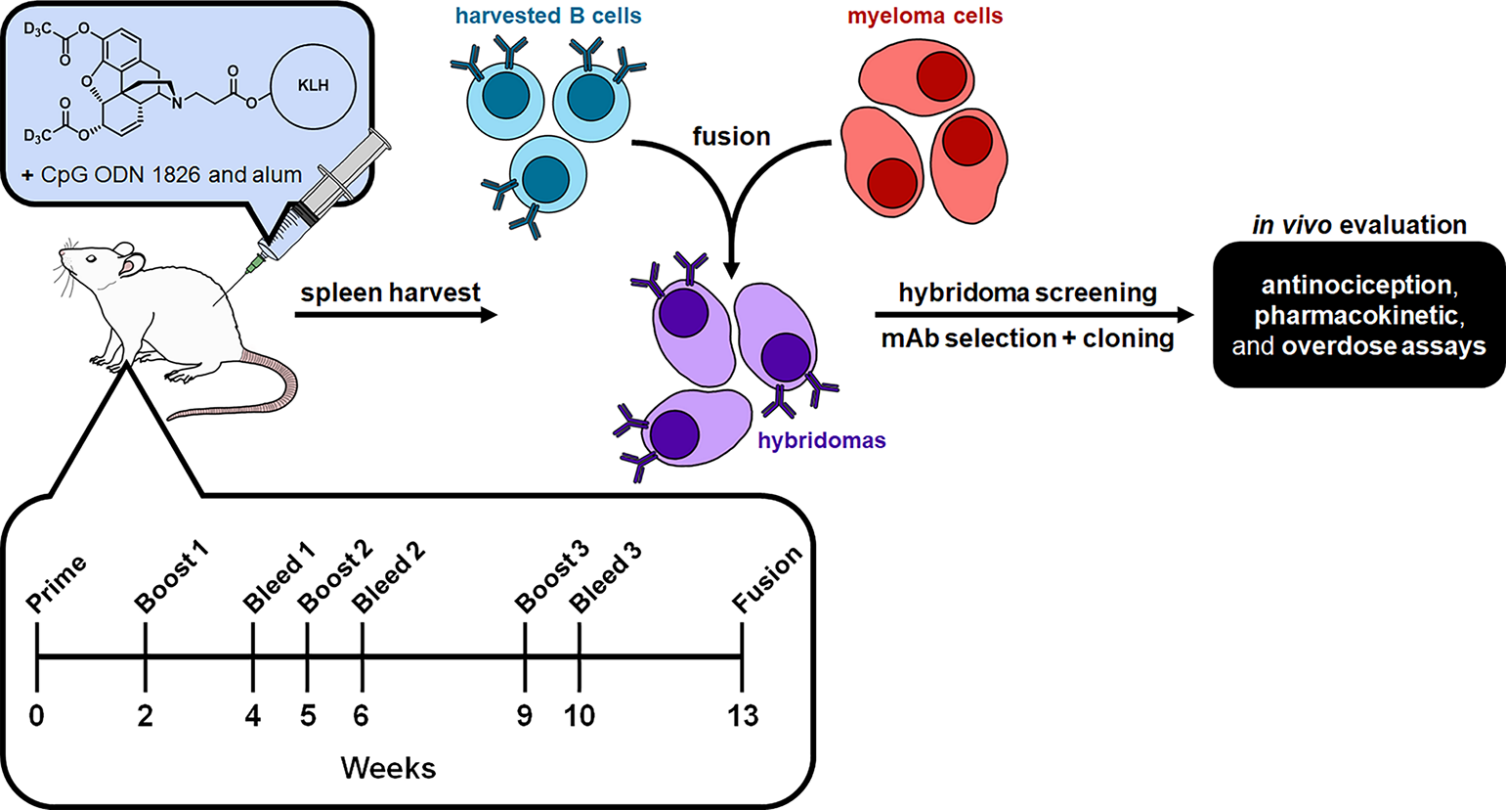
Overview of the research strategy and timeline for this study. The heroin hapten was conjugated to keyhole limpet hemocyanin (KLH) and formulated with an adjuvant cocktail consisting of CpG oligodeoxynucleotide (ODN) 1826 and alum to produce the heroin vaccine. The mice were immunized according to the timeline, and spleens were harvested after sufficient antibody generation. Isolated splenocytes were fused with myeloma cells to form hybridomas, which were screened using enzyme-linked immunosorbent assay and surface plasmon resonance for optimal monoclonal antibody (mAb) generation. Final purified mAbs were evaluated in murine models in antinociception, pharmacokinetic, and overdose assays against heroin and its metabolites.

To produce an assortment of mAbs to heroin and its metabolites, we chose to implement a deuterated heroin hapten (H_dAc_, Figure 1), which has been shown to elicit antibodies with nanomolar affinity to both heroin and 6-AM, leading to significant protective effects against drug antinociception and biodistribution.^22^ In brief, the hapten was conjugated to keyhole limpet hemocyanin (H_dAc_-KLH) and formulated with an adjuvant cocktail consisting of CpG oligodeoxynucleotide 1826 and alum. Mice were inoculated with this vaccine, and sera were collected on weeks 4, 6, and 10 to characterize the antibodies generated (Figure 1). Antibody titers were analyzed by enzyme-linked immunosorbent assay (ELISA) against the hapten conjugated to bovine serum albumin (H_dAc_-BSA). Antibody affinity to heroin and its metabolites, 6-AM and morphine, was observed by an SPR competitive binding assay using sera incubated with varying concentrations of drug that was flowed across a chip coated with H_dAc_-BSA. As anticipated, this vaccine produced robust antibody titer levels with nanomolar affinity to heroin and its metabolites (Table S1). This sequence of events led to the utilization of the mouse with the best antidrug serum profile for mAb production. This was achieved by harvesting the mice spleens to isolate B-cells for myeloma cell fusion to produce hybridomas. Successful fusion was confirmed by plating and screening hybridomas by using ELISA against H_dAc_-BSA, which revealed 17 hybridomas that tested positive for drug-binding capabilities (Tables S2 and S3). These hybridomas were implemented into SPR competitive binding assays against heroin and its metabolites to test their affinities and specificities. Based on this profiling, four hybridomas were selected for additional rounds of subcloning and grown at a larger scale to obtain milligram quantities of mAbs.

Upon cloning and isolation of stable cell lines, the four selected mAbs were implemented into further *in vitro* testing using SPR to observe their binding to heroin and its metabolites (Table 1). Against heroin, 4G12 and 6E1 had similar affinities, with 11D12 exhibiting the best dissociation constant (*K*_D_). However, 4G12 showed promising binding profiles against 6-AM, followed by 6E1 then 11D12. Monoclonal 6B11 failed to produce any affinity against heroin and 6-AM but was the only mAb to exhibit affinity for morphine among the four antibodies. The IC_50_ values revealed 11D12, 4G12, and 6E1 could bind to heroin and 6-AM on a nanomolar level. Antibody 11D12 had the best IC_50_ value among the three mAbs for heroin, 3.7 and 10.5-fold greater than 4G12 and 6E1, respectively. On the other hand, 6E1 displayed the tightest binding to 6-AM at 2.47 nM, which was 6.3 and 7.3 times greater than 4G12 and 11D12, respectively. This *in vitro* analysis of these four mAbs demonstrated an array of antibodies that displayed unique characteristics by either having compelling binding profiles to heroin (11D12), 6-AM (6E1), morphine (6B11), or heroin and 6-AM (4G12). We viewed this diversity to be critical, as it would allow us to probe whether heroin, its metabolites, or the combination of both should be targeted in the development of effective immunopharmacotherapies.

**Table 1:**
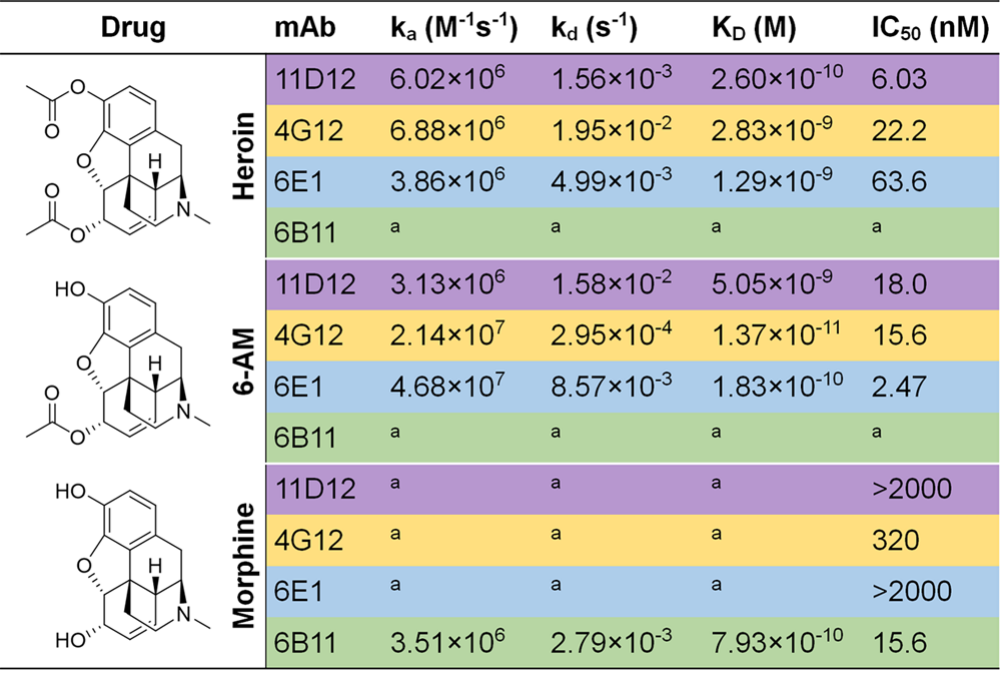
Binding Kinetics of Four Monoclonal Antibodies Against Heroin and Its Metabolites

a Not determined due to lack of binding.

### Antinociception Assessment

When heroin and its metabolites interact with the MOR, it results in euphoria, respiratory depression, and analgesia.^31,32^ Therefore, antinociception tests are often implemented to observe the effectiveness of potential therapeutics in affecting the perception of pain upon drug exposure. In these behavioral studies, nociceptive pain on the supraspinal level is detected using hot plate and spinal responses are observed through tail flick tests.^33^ This is accomplished by cumulatively dosing the animals with drug, while repeatedly assessing their response to thermal stimuli, until antinociception occurs at a predetermined time point to prevent tissue damage, which is considered 100% maximum possible effect of the drug.^34^ These repeated assessments generate dose–response curves that allow us to obtain the ED_50_ values, where half of the animals per group experience full antinociception. Antinociception tests are reliable in examining the effectiveness of antidrug antibodies, since the ability of the mAbs to interfere with MOR activation can be observed through the increase in ED_50_ values for treated mice.

**Figure 2:**
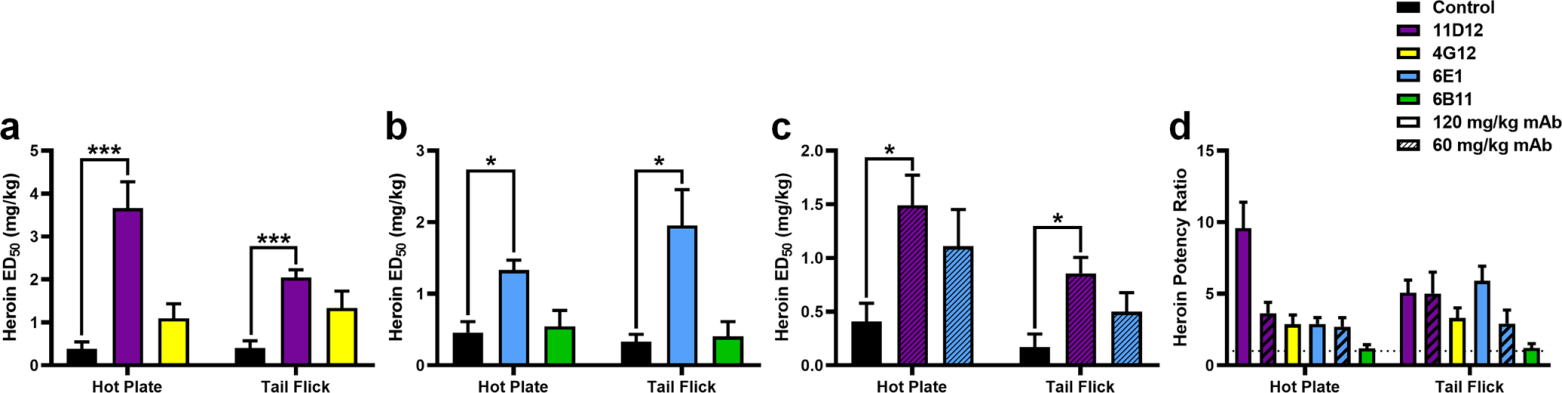
Antinociception assay results of mice administered different antibodies at high and low doses. (a,b) Hot plate and tail flick tests of mice injected IV with 120 mg/kg of mAb 30 min prior to cumulative IP injections of heroin. Calculated ED_50_ values for (a) 11D12 and 4G12 or (b) 6E1 and 6B11. (c) Antinociception test results using 60 mg/kg of 11D12 and 6E1. (d) Potency ratios were determined for each antibody group relative to control mice. Dashed lines denote control levels. Bars denote ± SEM in all plots; *n* = 6 per group. Significance is denoted by asterisks from one-way ANOVA with a Tukey’s post hoc test; *P < 0.05, ***P < 0.001 versus control group, nonsignificant analysis is not indicated. Abbreviation: ED_50_, effective dose; IP, intraperitoneal; IV, intravenously; mAb, monoclonal antibody; mg/kg, milligrams per kilogram.

The initial evaluation began by administering 120 mg/kg of each mAb to mice intravenously (IV), since that was the highest dose, which could be administered in a bolus injection. Mice were pretreated with mAbs 30 min prior to heroin exposure for antinociception studies. At the highest dose, 11D12 and 6E1 were significantly different from controls in hot plate and tail flick tests (Figure 2a, b). Monoclonals 4G12 and 6B11 showed no significant difference from controls in either examination. Thus, only 11D12 and 6E1 were implemented into additional antinociception studies at a lower dose to ascertain which mAb would be applied for further *in vivo* analyses.

The two most promising mAbs were administered at a lower dose of 60 mg/kg prior to cumulative drug exposure for antinociception studies. Even with the lower dose, 11D12 was still significantly different from controls in both hot plate and tail flick tests, while 6E1 failed to follow this trend (Figure 2c). These results were interesting, since it provided insight into immunotherapeutic prophylactic treatment; in sum, a high affinity antibody to heroin will render the metabolites ineffective, and broad-spectrum metabolite sequestering is not required. This is supported by the fact that 6B11 failed to produce any difference from control groups in hot plate and tail flick tests, yet was the only mAb that had affinity for morphine. Moreover, 4G12 demonstrated excellent affinity to both heroin and 6-AM but did not yield any significant differences. Finally, even though 6E1 possessed single digit nanomolar affinity to 6-AM, it did not protect against drug effects as well as 11D12, the mAb that had the preeminent binding affinity against heroin.

Having established antinociception data on the four mAbs, 11D12 was considered the best mAb candidate from the group not only because it showed significant differences in ED_50_ values from controls when administered at high and low concentrations but also since it had higher potency ratios than the other mAbs. Suffice it to say, potency ratios are an effective means to identify whether a immunopharmacotherapeutic can be considered beneficial for treating substance use disorders, since it has been proposed that an effective therapeutic should pass a “naltrexone benchmark”, which requires an 8-fold potency shift in behavioral studies.^35^ Moreover, 11D12 is the only mAb among the candidates that passed this benchmark and displayed significant differences from controls at both high and low levels of mAb treatment, thus it was chosen for further *in vivo* analyses.

### Rescue Antinociception Testing

Although the antinociception tests showed the significant protective effects of 11D12 when administered prior to drug exposure, a critical aspect of passive immunization that active immunization cannot provide is the ability to be administered as a therapeutic post-drug exposure to reverse deadly drug effects elicited by MOR activation. Naloxone is an FDA-approved small-molecule antagonist that is commonly used to reverse such lethal opioid effects. However, its short half-life has proved problematic in the current opioid epidemic due to “renarcotization”, where naloxone needs to be administered repeatedly to be effective against fatal opioid effects.^36^ Passive immunization of mAbs would thwart renarcotization since antibodies have longer half-lives and exhibit similar effects of pharmacokinetic antagonism.^8,12,37^

To mimic realistic clinical applications of using a mAb to treat drug toxicity, we conducted rescue antinociception tests to observe whether 11D12 could reverse drug effects after a full antinociceptive dose. For this study, baseline nociception responses of mice were recorded, then mice were administered a 3 mg/kg dose of heroin intraperitoneally (IP), which was approximately 8-fold greater than the ED_50_ of heroin in naïve mice (Figure S1). After 15 min, nociception responses were recorded again, then mice were injected with 120 mg/kg mAb IV and observed for an additional hour. Based on the drug affinity and antinociception assays, *vide supra*, 11D12 and 4G12 were examined. Recourse here dictated the use of 11D12, as it was the best candidate from the previous antinociception study with the greatest affinity for heroin (Figure 2), while 4G12 presented the best affinity for 6-AM (Table 1). One might question this logic, as 4G12 did not perform well in the previous antinociception study; however, we wanted to implement it as a comparison point, since the importance of heroin metabolites could greatly differ depending on pre/post antibody treatment.

**Figure 3:**
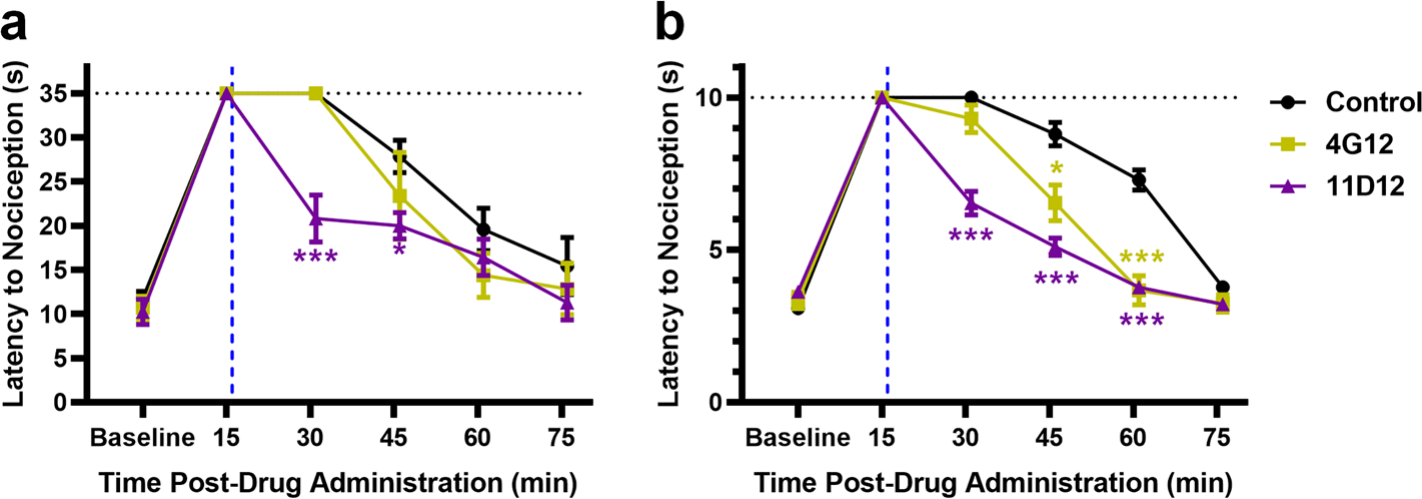
Rescue from heroin-induced antinociception. After baseline nociception was measured, mice were administered 3 mg/kg of heroin IP, and nociception was measured again 15 min postdrug exposure. After hot plate (a) and tail flick (b) tests mice were administered, 120 mg/kg of either 11D12 or 4G12 IV at 16 min postdrug exposure (blue dashed line). Nociception was measured at 15 min intervals until all mice returned to baseline. Black dashed lines denote the antinociception cutoff times, where the drug is at 100% maximum possible effect. Blue dashed lines denote administration of the antibody. Data points denote the means ± SEM; *n* = 6 per group. Significance is denoted by asterisks from two-way RM ANOVA with Dunnett post hoc test; *P < 0.05, ***P < 0.001 versus control group, nonsignificant analysis is not indicated. Abbreviation: IP, intraperitoneal; IV, intravenously; min, minutes; s, seconds.

Under conditions detailed, *vide supra*, mAb 11D12 produced significant differences from controls in returning mice exposed to heroin back to baseline starting at 30 min postdrug exposure or 15 min post-mAb administration (Figure 3). Interestingly, mAb 4G12 only showed significance in tail flick tests starting at 45 min postdrug exposure but was able to bring mice back to baseline faster than controls, similar to 11D12 (Figure 3b). These results further reinforce the simple antinociception data, *vide supra*, wherein an antibody targeting heroin is important for early intervention rather than 6-AM, which has been considered the main active metabolite of interest to inhibit for effective therapeutic effects.^21,29,38^

### Pharmacokinetics

To observe how the selected mAbs alter heroin’s metabolism *in vivo*, we conducted pharmacokinetic studies by injecting naïve mice IV with either drug, mAb, or drug and mAb. For the drug and mAb combination, mice were treated with either 4G12 or 11D12 half an hour before they were injected with 1 mg/kg of heroin. For all groups, mice were repeatedly sampled retro-orbitally at various time points, and samples were analyzed by LC-MS/MS (Figure S2). Pharmacokinetic analysis was done using a two-compartmental model since drugs and mAbs were injected IV using a bolus dose. Both 11D12 and 4G12 were utilized in this study so that differences could be noted in the pharmacokinetics between mAbs that had higher affinity for either heroin or 6-AM. In theory, the half-life of the drug should be extended if the mAb binds tightly to the drug or metabolite, based upon formation of a stable antibody-drug complex.^13^

When mice were injected with only drug, heroin and 6-AM had half-lives of 0.7 and 8.9 min, respectively (Table 2). The half-life of mAb 11D12 was 14.7 days, while mAb 4G12 was almost double at 27.6 days. In the presence of 4G12, the half-life of heroin was not altered, but the half-life of 6-AM was extended to 16.2 h, almost 110-fold greater than the metabolite alone. On the other hand, 11D12 extended the half-life of heroin to 0.68 h, approximately 58 times longer than the original half-life of the drug. Furthermore, in the presence of 11D12, 6-AM had a half-life of 1.96 h, which is about 13-fold more than its initial value.

**Table 2:** Pharmacokinetics of Heroin and 6-AM in the Absence and Presence of Monoclonal Antibodies

Analyte		*t*_1/2_	*C*_max_	AUC_0-∞_
Heroin		0.70 min	257.75 ng/mL	261.07 min·ng/mL
6-AM		8.89 min	1283.4 ng/ML	7192.8 min·ng/ML
4G12		27.6 days	940.91 μg/mL	26877 days·μg/mL
11D12		14.7 days	839.60 μg/mL	11417 days·μg/mL
4G12	Heroin	a	a	a
	6-AM	16.2 h	1191.1 ng/mL	14624 h·ng/mL
11D12	Heroin	0.68 h	1345.9 ng/mL	444.42 h·ng/mL
	6-AM	1.96 h	594.11 ng/mL	222.22 h·ng/mL

a No values recorded due to limited data points for pharmacokinetic analysis. Amount of drug detected at the time point was the same as heroin only analysis.

These results corroborate the significant protective effects of 11D12 observed in the antinociception studies *vide supra*. Although *in vitro* studies showed 4G12 had some affinity for heroin (Table 1), the pharmacokinetic study revealed that this mAb had little ability to alter the half-life of the drug and mainly affected 6-AM. Conversely, 11D12 was able to interact with both heroin and 6-AM, thus we were able to detect both drugs at prolonged time points in mice, resulting in the extended half-lives. However, 11D12 does not interact with 6-AM as strongly as 4G12, as seen in both the *in vitro* and pharmacokinetic analyses, yet had better protective effects in both prophylactic and rescue antinociception studies.

### Lethality Challenge

**Figure 4:**
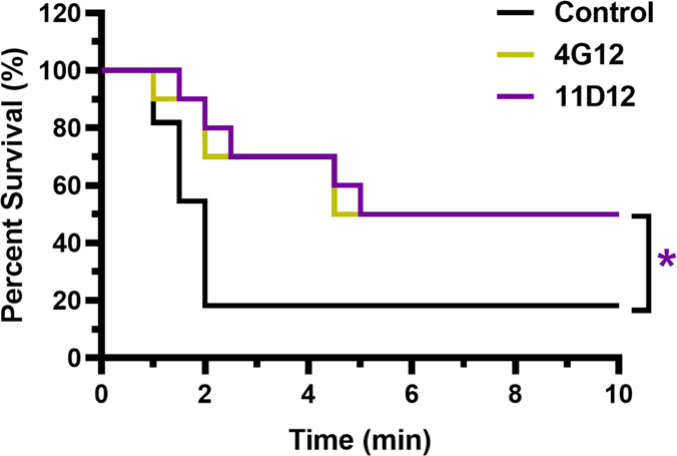
Protection from lethal doses of heroin. Mice (*n* = 10) were injected with 120 mg/kg of 11D12 or 4G12 IV 1 h before being dosed with 30 mg/kg of heroin IV to observe the mAbs’ abilities to protect against lethal doses of heroin. Time to death was recorded until death no longer occurred. Kaplan–Meier survival curve. Significance is denoted by an asterisk from log-rank test comparing each antibody group to control; *P < 0.05 versus control group, nonsignificant analysis is not indicated. Abbreviation: IV, intravenously; min, minutes.

Having established on multiple levels that heroin is the key chemical entity to target while using an antibody, we next sought to probe this in a lethality challenge. Again, 11D12 would be engaged based upon its performance in the antinociception and rescue studies, and to keep this trial consistent with the rescue experiment, 4G12 was also included as a comparison tool. To conduct this study, mice were prophylactically treated with 120 mg/kg of mAb IV an hour before they were administered 30 mg/kg of heroin IV, which is approximately 80-fold greater than the ED_50_ of heroin in naïve mice. At this dose of drug, heroin is at an almost 51-fold excess of mAb doses and half the control mice die within 1.5 min. This experiment indicated mAb 11D12 could significantly protect against lethal doses of heroin, while 4G12 did not produce any significant results (Figure 4). Although 4G12 did produce similar levels of survival as 11D12 at the end of the experiment, 11D12 exhibited a higher percentage of survival at earlier time points than 4G12, thus the difference in significance. Both antibodies had 30% greater survival rates than control.

Lethality associated with opioid overdose is mostly due to respiratory depression. In our experiment, most animals in the control group lost consciousness almost immediately and rarely had intense seizures. However, the groups treated with the mAbs exhibited different symptoms post-lethal drug exposure. Mice treated with both mAbs suffered from clonic seizures at varying levels. Most mice began with forelimb clonus and loss of posture, which later advanced into running bouncing clonus and tonic-clonic seizures. Death would occur shortly after tonic hindlimb extension. These symptoms were surprising, since heroin and 6-AM have not been reported to cause seizures; however, myoclonus and seizing have been reported with high doses of morphine in humans.^39−41^ Since morphine is the final metabolite of heroin and the dose examined was in 51-fold excess of the mAb concentration, it is possible the heroin or 6-AM not bound to antibody is converted to morphine and the large presence of this metabolite caused the severe seizing activity.

Although most mice that were administered mAb and the lethal dose exhibited some seizing activity, there were distinct differences between the two antibody groups (Table S4). We observed that mice treated with 11D12 would either advance very quickly through the different seizure types and expire or remain in forelimb clonus, then begin walking in circles, the latter of which is normal behavior observed in mice exposed to opioids. Generally, mice treated with 11D12 would remain very active, if they survived. However, for 4G12, the mice tended to progress from forelimb clonus and loss of posture to running bouncing clonus, then revert to the previous stage and progress forward again. These mice cycled through the different seizure scales repeatedly until they eventually died after tonic hindlimb extension, or if they survived, they remained motionless and inactive. In addition to 11D12 significantly protecting against lethal doses of heroin, its ability to produce less severe cycling through seizures was noteworthy.

## Conclusions

For decades, research has focused on developing a vaccine with broad-spectrum protection against heroin’s metabolites,^18−22^ since studies showed 6-AM and morphine were the major culprits in eliciting the common psychoactive and lethal effects of heroin use.^29,42,43^ However, by utilizing mAbs with unique binding profiles to heroin and its metabolites, our study exposes heroin as the principal drug target for developing an effective immunotherapeutic. Using this strategy, a mAb was discovered that can protect against the psychoactive and fatal effects of heroin by specifically inhibiting the parent drug structure. Moreover, this is the first report of a mAb that can significantly protect against lethal doses of heroin and suggests a necessary paradigm shift for future development of effective immunopharmacotherapy against heroin. Finally, although the metabolic complexity and opioid receptor recognition that heroin possesses as a drug had manifested into a complex and challenging immunologic target for vaccine development, the research we have detailed is expected to facilitate the preparation of improved heroin vaccines, thus enhancing opportunities for clinical advancement of immunopharmacotherapy.

## Supporting Information Available

Experimental materials and methods of chemical, biochemical, and *in vivo* procedures. Supplementary tables and figures of end point titers, hybridoma characterization, dose optimization for behavior experiments, and pharmacokinetic sample analyses (PDF)

## Abbreviations

6-AM: 6-monoacetylmorphine
BSA: bovine serum albumin
ELISA: enzyme-linked immunosorbent assay
KLH: keyhole limpet hemocyanin
IP: intraperitoneally
IV: intravenously
mAb: monoclonal antibody
MOR: μ-opioid-receptor
OUDs: opioid use disorders
SPR: surface plasmon resonance
