# Supplementary material for: Development of an Effective Monoclonal Antibody against Heroin and Its Metabolites Reveals Therapies Have Mistargeted 6-Monoacetylmorphine and Morphine over Heroin
Source: ACS Cent Sci. 2022 Oct 6;8(10):1464–70. doi: 10.1021/acscentsci.2c00977 (PMC9615117; doi:10.1021/acscentsci.2c00977)
Supplement: Supplementary file 1 — oc2c00977_si_001.pdf [file oc2c00977_si_001.pdf]

Supporting Information

Development of an effective monoclonal antibody against heroin and its metabolites reveals therapies have mistargeted 6-monoacetylmorphine and morphine over heroin

Authors:

Jinny Claire Lee[a], Lisa M. Eubanks[a], Bin Zhou[a], and Kim D. Janda[a, *]

Affiliations:

[a] Departments of Chemistry and Immunology, The Skaggs Institute for Chemical Biology, Worm Institute for Research and Medicine (WIRM), The Scripps Research Institute, 10550 North Torrey Pines Road, La Jolla, California 92037, United States

*** To whom correspondence should be addressed:** Kim D. Janda; kdjanda@scripps.edu; Tel.: +1-858-784-2516; Fax: +1-858-784-2595

Table of Contents

Materials and Methods 1

Safety statement 1

Chemistry 1

Synthesis and bioconjugation of the deuterated heroin hapten 1

Biochemical and in vivo procedures 1

Animals 1

Drugs 1

Vaccinations and hybridoma generation 2

Enzyme-linked immunosorbent assay (ELISA) 2

Surface plasmon resonance (SPR) 2

Antinociception 5

Pharmacokinetics 6

Analyses of blood samples 8

Lethality heroin challenge 8

Supplementary Tables and Figures 10

Table S1. Endpoint titers during mouse immunizations 10

Table S2. Hybridoma titers and inhibitive binding profiles. 10

Table S3. Hybridoma titer and isotype characterization. 12

Table S4. Statistical analysis of heroin-induced symptoms. 13

Figure S1. Optimization of drug dose for rescue antinociception assay. 14

Figure S2. Quantification of drug and monoclonal antibody concentrations at various timepoints for pharmacokinetic analyses 15

References 16

Materials and Methods

Safety statement

No unexpected or unusually high safety hazards were encountered.

Chemistry

Synthesis and bioconjugation of the deuterated heroin hapten

The deuterated heroin hapten (H_{dAc}) was synthesized according to previously published procedures.[1] Conjugation to keyhole limpet hemocyanin (KLH) and bovine serum albumin (BSA) were completed following previous studies.[1]

Biochemical and in vivo procedures

Animals

6-week-old male Swiss Webster mice were obtained from Taconic Farms (Germantown, NY, USA) and allowed to acclimate for approximately 1 week before antinociception, pharmacokinetic, and overdose studies. All animal studies were performed in compliance with the Scripps Institutional Animal Care and Use Committee (Protocol #08-0127) and were in accordance with the National Institute of Health *Guide for the Care and Use of Laboratory Animals*. Mice were group-housed in an AAALAC-accredited vivarium containing temperature- and humidity-controlled rooms, with mice kept on a reverse light cycle (lights on: 9PM to 9AM). All experiments were performed during the dark phase. General health was monitored by both the scientists and veterinary staff at The Scripps Research Institute.

Drugs

Heroin hydrochloride (HCl) was obtained from the National Institute on Drug Abuse as a solid. Opioids for cross-reactivity screening and deuterated drug standards were obtained from Cerilliant as solutions in organic solvent. Heroin HCl was dissolved and diluted into saline for animal administration.

Vaccinations and hybridoma generation

Each female A/J mouse received 100 µL of a vaccine formulated with 50 µg of H_{dAc}-KLH, 50 µg of CpG ODN 1826 (Eurofins MWG Operon), and 50 µg of alum (Alhydrogel®, Invivogen) in PBS pH 7.4. Vaccines were made fresh and mixed for at least 1 hour, prior to injection. The suspension (100 µL per mouse) was administered intraperitoneally (IP) on weeks 0, 2, 5, and 9. No adverse reactions were observed. Blood sampling was performed on weeks 4, 6, and 10. A final intravenous (IV) infusion of conjugate was performed on week 13. Three days later, one animal was sacrificed, and its spleen was extracted, homogenized and splenocytes were washed with RPMI medium. The resulting B-cells were fused with X63Ag8.653 nonproducing myeloma cells using PEG1500 and selected by culturing in HAT medium. Of the plated hybridomas, 47 were positive for hapten binding by ELISA and further screened by Biacore. Of the 47 hybridomas screened, 17 were frozen and the best were selected for two rounds of subcloning by limiting dilution (4G12, 6B11, 6E1, 11D12). All monoclonal antibodies (mAbs) were isotyped.

Enzyme-linked immunosorbent assay (ELISA)

For initial screening of mouse serum and hybridoma supernatants, a similar procedure was used for end point titer determinations as previously reported.[2] For isotyping, goat antimouse antibodies recognizing kappa/lambda LC or gamma 1/2a/2b HC were used for plate coating.

Surface plasmon resonance (SPR)

1) *Ranking antibody binding affinity and specificity from hybridoma culture supernatant by competitive SPR analyses*

   47 hybridomas were positive for hapten binding in ELISA and were further analyzed by SPR to rank drug affinities and specificities against 6-AM, heroin, and morphine. The analyses were conducted on a Biacore 3000 instrument equipped with a CM5 sensor chip (Cytiva). The ligand, deuterated heroin hapten, was immobilized onto the chip surface using Amine Coupling Kit (Cytiva) as follows: 1) flow cell (Fc) 2 surface was activated for 7 minutes with a 1:1 mixture of 0.1 M NHS and 0.4 M EDC at a flow rate of 10 µL/min. 2) The deuterated heroin hapten resuspended in 10 mM sodium acetate (pH 4.0) were immobilized at a density level of 2,500 resonance unit (RU) on Fc2; whereas Fc1 was immobilized with BSA (resuspended in 10 mM

sodium acetate, pH 4.0) at a similar density level to serve as reference surface. 3) All surfaces were blocked with 7-minute injection of 1.0 M ethanolamine-HCl (pH 8.5). All binding assays were conducted at a flow rate of 30 µL/min at 25 °C, using 1X HBS-EP+ buffer (Cytiva) as running buffer. Hybridoma culture supernatant was individually diluted and pre-incubated with various compounds (heroin at 15.6 nM, 6-AM at 150 nM, and morphine at 250 nM) at room temperature for 1 hour before the mixture was injected over the sensor chip surface to observe the binding. For each analysis cycle, one pre-incubated sample was injected for 300 sec over all flow cells (i.e., Fc1 through Fc2), followed by 150 sec of dissociation in running buffer. The relative response at the end of dissociation (reference flow cell subtracted, i.e., Fc2 minus Fc1) was recorded in the sensorgram and was used for inhibitive binding evaluation. After each sample analysis, all flow cells were regenerated with 30 seconds injection of 10 mM Gly-HCl, pH 1.5, before next cycle of analysis. From this analysis, 17 hybridomas were selected for more in-depth *in vitro* competitive analyses, and 6 hybridomas were selected for subcloning and mAb production at the end.

2) *Determine IC50 of heroin, 6-AM and morphine for selected mAbs using SPR methodology*

Six selected mAbs were subjected to more in-depth competitive analyses to determine the IC50 of heroin, 6-AM and morphine compounds. In brief, the chip was prepared *vide supra*. Each mAb was pre-titrated and pre-incubated with 12 different concentrations of compounds (two-fold dilution from 2000 nM to 1.95 nM with no drug control for heroin, 6-AM and morphine) at room temperature for 1 hour, before the mixture was injected over the sensor chip surface to observe the binding. For each analysis cycle, one pre-incubated sample was injected for 300 sec over all flow cells (i.e., Fc1 through Fc2), followed by 150 sec of dissociation in running buffer. The relative response at the end of dissociation (Fc2 minus Fc1) was recorded in the sensorgram and was used for inhibitive binding evaluation. After each sample analysis, all flow cells were regenerated with 30 seconds injection of 10 mM Gly-HCl, pH 1.5, before next cycle of analysis. The IC50 was calculated using GraphPad Prism software (ver. 6.00).

3) *Determine the binding kinetics for selected mAbs via SPR*

The binding kinetics of heroin, 6-AM, and morphine towards selected mAbs were conducted on a Biacore S200 instrument equipped with a series S CM5 sensor chip (Cytiva). Each selected mAb was immobilized on the CM5 chip surface using Amine Coupling Kit (Cytiva) as follows: 1) The active flow cell Fc 2 or Fc4 surface was activated for 7 minutes with a 1:1

mixture of 0.1 M NHS and 0.4 M EDC at a flow rate of 10 µL/min; 2) Individual mAb was resuspended in 10 mM sodium acetate (with pre-determined pH per pH scouting results) and injected over activated Fc2 or Fc4 at 10 µL/min aiming at immobilization level of 1500 RU; 3) The flow cell surfaces were blocked with a 7-minute injection of 1.0 M ethanolamine-HCl (pH 8.5) at a flow rate of 10 µL/min. As a reference flow cell, Fc1 or Fc3 was activated with NHS/EDC and blocked with ethanolamine as described above. The kinetics were determined via single cycle kinetics methodology with five analyte concentrations (ranging from 10 nM to 2000 nM), and the data was fitted using a 1:1 binding model. The assay was run in PBS-P+ buffer (Cytiva) at a flow rate of 50µL/min with a 10 Hz data collection rate as follows: 1) 3 x startup cycles (each cycle includes 30 sec of running buffer injection and 300 sec of dissociation, all at a flow rate of 50 µL/min, the chip surface was regenerated with Gly-HCl (pH 1.5) for 30 sec) were conducted before SCK analysis. 2) For binding kinetics, heroin, 6-AM or morphine were prepared in running buffer in five different concentrations. The diluted compound samples were then injected for 30 sec consecutively from low to high followed by 3600 sec of dissociation in running buffer. 3) The sensor chip surface was regenerated with 30 sec of injection of Gly-HCl (pH1.5) solution before the next cycle of SCK analysis. A blank running buffer injection was also conducted before each compound run using the exact same conditions for SCK analysis. All data, including responses from Fc2 minus Fc1 or Fc4 minus Fc3, were collected by Biacore control software in a result file. The run data sets stored in the result file were then analyzed by Biacore S200 evaluation software (ver. 1.1 build:27) using predefined LWM kinetics/affinity single-cycle method. Each run has passed quality control as determined by the Biacore S200 evaluation software, which includes: 1) whether or not the kinetic constants are within instrument specifications; 2) whether or not the kinetics constants appear to be uniquely determined; 3) whether or not significant bulk contribution is found.

4) *SPR quantification of selected mAbs in sera for pharmacokinetics studies*

To quantify the amount of administrated mAb left in the serum over time, the sera collected at various time points were analyzed via SPR binding assay, and the mAb concentration of each time point was interpolated from a standard mAb binding curve. In brief, the quantitative assay was conducted on a Biacore 3000 instrument equipped with a CM5 chip. A chip was immobilized with deuterated heroin hapten *vide supra*. For each tested mAb, a standard mAb

binding curve was created by injecting a known concentration sample (covering 50, 100, 200, 400, 800, 1600, 3200 and 6400 ng/mL) over all flow cells (i.e., Fc1 through Fc2), followed by 150 sec of dissociation in running buffer. The relative response at the end of dissociation (reference flow cell subtracted, Fc2 minus Fc1) was recorded in the sensorgram and was used for standard curve fitting. After each sample analysis, all flow cells were regenerated with 30 seconds injection of 10 mM Gly-HCl, pH 1.5, before next cycle of analysis. The unknown sample from each time point was prepared in running buffer using pre-determined dilution factor and was injected over both Fc1 and Fc2 as described above. The relative response at the end of dissociation was used to interpolate the serum mAb concentration from the fitted standard curve using GraphPad Prism software (ver. 6.00).

Antinociception

Nociception was measured in two behavioral tests, hot plate (supraspinal) and tail flick (spinal), as previously described.[3] In the hot plate test, mice were placed in an acrylic cylinder (14 cm in diameter x 22 cm in height) on a 55 °C surface, and the latency to perform one of the following nociception responses: hind paw licking, hind paw withdrawal/shaking, or jumping was timed with a 35 s maximum cutoff time to prevent tissue damage. The tail flick test was performed using an ITC Life Science Tail Flick Analgesia Meter to aim a high-intensity light beam (45% active intensity) at the tail. The latency to tail withdrawal from the beam was timed with a 10 s maximum cutoff time to prevent tissue damage. Since, tail flick is a more reflective behavior, the hot plate test was performed first, followed by the tail flick test. Mice were baselined once prior to recording baseline measurements to acclimate them to the testing environment. Baseline measurements for each test were recorded prior to drug injection. All antinociception tests used 7-week-old male Swiss Webster mice.

During cumulative dosing antinociception, mice (n = 6/group) received increasing amounts of drug over the course of the study. Prior to drug exposure, mice were inoculated with mAbs 30 minutes before baseline measurements were conducted. Immediately after obtaining baseline values for both tests, heroin HCl was administered IP and latency to nociception was measured 15 min post-injection. Testing was repeated in 15 min intervals until full antinociception was observed in both assays (i.e. maximum cutoff time was reached). Both drugs were tested at the following intervals to generate a full dose-response curve: 1, 2, 3, 5, and 9 mg/kg. Antinociception

data were transformed from time to percent maximum possible effect (%MPE), which is calculated as: $\%MPE = \frac{(\text{test} - \text{baseline})}{(\text{cutoff} - \text{baseline})} \times 100$

These data were then fit using a log(agonist) vs. normalized response nonlinear regression in GraphPad PRISM 8. The ED_{50} values were determined for each antinociception test and individual treatment groups, and subsequently used to determine potency ratios. Data were tested for statistical significance in antinociception by using one-way ANOVA with a Tukey's post hoc test.

During rescue antinociception, mice (n = 6/group) received a bolus dose IP of 3 mg/kg heroin HCl after baseline measurements were performed. After 15 minutes post-drug exposure, mice were tested on the hot plate and tail flick apparatus. At 16 minutes post-drug exposure, mice were inoculated with mAb and tested using both antinociception assays every 15 minutes until they returned to baseline values. These data were graphed using the time post-drug administration in minutes vs. latency to nociception time in seconds in GraphPad PRISM 8. Data were tested for statistical significance by using two-way repeated measures ANOVA with Dunnett post hoc test.

Pharmacokinetics

Naïve 7-week-old male Swiss Webster mice were injected IV with a solution containing 0.29 mg/mL of heroin HCl (n = 6 - 8) in order to provide a dose of 1 mg/kg for drug only and mAb with drug pharmacokinetic analyses. For mAb pharmacokinetic analysis, mice received 60 mg/kg doses of mAb IV in the opposite eye vein. For mAb and drug pharmacokinetics, mice were administered mAb 30 minutes before drug injections. The animals were returned to their home cage and each animal had blood taken retro-orbitally at independent, preset periods of time to generate n = 3 - 4 data points for each measured time point. For drug only pharmacokinetic analysis, samples were collected at t = 30 sec, 3 min, 10 min, 15 min, 30 min, 45 min and immediately added to formate buffer in a 1:1 ratio. For drug and mAb pharmacokinetic analysis, samples were collected at t = 3, 15, 30, 60, 180, 360, 1140 min and immediately added to formate buffer[4-6] (10 mM ammonium formate buffer pH 3.0, 8 mg/mL NaF) in a 1:1 ratio. For mAb only pharmacokinetic analysis, samples were collected at t = 5 min, 30 min, 3 hr, 6 hr, 1, 2, 3, 4, 6, 8, 10, 15, 20, 25, 30, 35 day and centrifuged at 13,000 rpm for 15 min to collect sera. The sera was stored at –20 °C until analysis by SPR, explained *vide supra*. All collected blood samples were stored on ice until analysis. Samples for drug only and drug with mAb were immediately processed for LC-MS/MS analysis as explained *vide infra*.

On the day of analysis, samples were left on ice to minimize heroin deacetylation to 6-AM. 50 µL of whole blood:formate buffer (1:1 ratio) was pipetted into a fresh 1.5 mL microcentrifuge tube. 14 µL of acetonitrile (ACN), 24 µL of 0.7 µg/mL deuterated heroin in ACN, 24 µL of 0.7 µg/mL deuterated 6-AM in ACN, and 250 µL of ACN/MeOH (85/15) were added to the tube. The deuterated drug that was added to the sample was an internal standard used to aid in LC-MS/MS analysis. After vigorously vortexing the mixture for 30 sec, the samples were centrifuged at 4,500 rpm for 10 min at 4 °C. The top solvent layer was removed and transferred to a new tube and evaporated for 1.5 hr using Genevac (low BP-mix, 40 °C). The resulting residues were dissolved in 32 µL of HPLC grade ACN and sonicated for 3 minutes to ensure all compounds were resuspended. The sample was centrifuged at 10,000 rpm for 5 min at 4 °C. The solution was moved to LC-MS vials for analysis. LC-MS/MS analysis of samples and interpolation of drug concentrations in samples are explained *vide infra*.

Pharmacokinetic analyses were conducted using the two-compartmental model since all mice received IV bolus injections. Elimination and distribution phases were determined using established two-compartmental analysis, where equations were utilized to determine half-lives ($t_{1/2}$), maximum concentrations (C_{max}), and area under the curve (AUC).[7-9] The initial concentration or C_{max} was determined by adding the inverse natural log of the distribution (A) and elimination phase (B) y-intercepts together. The elimination rate constant (β) was calculated by determining the slope of the natural log of drug concentration over time during the elimination phase. The half-life was calculated using the formula:

$$t_{\frac{1}{2}} = \frac{ln\,(2)}{\beta}$$

The AUC was determined using the following equation:

$$AUC = \frac{A}{\alpha} + \frac{B}{\beta}$$

where A is the slope of the natural log of drug concentration over time during the distribution phase and all other variables were used in equations explained previously.

Analyses of blood samples

Standard curve samples were prepared by following the same sample processing method as experimental blood samples *vide supra*, but sample preparation consisted of substituting the 14 µL

of ACN with spike drug in ACN. The final concentrations of spiked heroin in samples were 1, 8.75, 17.5, 87.5, 175, 875, 1750, and 8750 ng/mL. The final concentrations of spiked 6-AM in samples were 10, 22, 44, 88, 175, 875, 1750, 8750 ng/mL.

All samples were submitted for LC-MS/MS analysis and were run on an Agilent 6135 single quadrupole mass spectrometer, coupled to an Agilent 1260 LC stack using a Agilent SB-C8 300A 4.6 × 50 mm column. For the mobile phase, water/0.1% formic acid (A) and ACN/0.1% formic acid (B) were used. The gradient started at 90% (A) then increased to 95% (B) in 6 min, and held there for 4 min. The total run time was 10 min. The flowrate was set to 0.5 mL/min and the sample injection volume was 5 μL. Ions with mass corresponding to heroin (*m/z* 369.1 → 370.1, ESI+), 6-AM (*m/z* 327.1 → 328.1, ESI+), heroin-d_9 (*m/z* 378.2 → 379.2, ESI+), 6-AM-d_3 (*m/z* 330.1 → 331.1, ESI+), were extracted and the resulting peaks were integrated.

Samples were plotted as relative responses against known concentrations of drug in GraphPad PRISM 8. Relative responses were obtained by dividing the signal area of drug over the signal area of the deuterated drug. The standard curve was generated by least-squares regression with a weighting factor of $1/x$. Drug concentrations in experimental samples of blood were quantified by interpolating using the standard curve.

Lethality heroin challenge

Naïve 7-week-old male Swiss Webster mice were injected IV with 120 mg/kg of mAb an hour before drug exposure. A solution containing 8.3 mg/mL of heroin HCl (n = 10) was injected IV into the opposite eye vein, in order to provide a dose of 30 mg/kg. The mice were continuously monitored until 6 hours post-drug exposure. The time to death was recorded when both loss of breathing and reflex was observed.

Seizing activity was recorded in tandem to lethality scoring and was based on a 5-point scale: head nodding/twitches, forelimb clonus/writhing/loss of posture, running bouncing clonus, tonic hindlimb extension, time of death. When a mouse cycled through different seizing activity continuously, the criterion was marked with a tally to indicate the number of times the mouse cycled through the scale. To measure the effect of monoclonal antibody treatment on heroin-induced effects, the appearance of overdose symptoms (death or seizures) was assigned a value of incidence (present = 1, absent = 0) for each mouse regardless of time of symptom onset. Group

data was analyzed via the χ^2 distribution. The theoretical frequencies for each mAb group for seizures and death were based on the drug only control group.

Supplementary Tables and Figures

Table S1. Endpoint titers during mouse immunizations.

Titers were determined by ELISA against H_{dAc}-BSA.

Mouse	Week 4	Week 6	Week 10
1	25600-51200	12800-25600	25600-51200
2	25600-51200	51200-102400	25600
3	25600-51200	51200-102400	25600-51200
4	25600-51200	25600	25600
5	25600-51200	102400	51200-102400

Table S2. Hybridoma titers and inhibitive binding profiles.

47 hybridomas were positive for hapten binding and were analyzed by SPR to observe drug affinities to 6-AM, heroin, and morphine. No inhibition scores recorded are indicated with an asterisk (*). From this analysis, 17 hybridomas were selected for more in-depth *in vitro* analyses.

No.	Name	H_{dAc}-BSA Titer	6-AM (15.6 nM)	Heroin (125 nM)	Morphine (250 nM)
1	1D8	211.44	inferior	inferior	inferior
2	1E11	2820.81	*	*	*
3	2D9	21.3	strong	strong	*
4	2E3	840.52	*	*	*
5	3A8	-5.6	*	*	*
6	3B8	-0.6	*	*	*
7	3D10	-7.25	*	*	*
8	3E12	14.63	strong	strong	*
9	3H4	-5.37	*	*	*
10	4A3	71.76	*	*	*
11	4G12	111.15	strong	strong	strong
12	5A3	-8.11	*	*	*
13	5C5	27.21	strong	moderate	inferior
14	5D6	117.83	moderate	moderate	*
15	5E7	-6.64	*	*	*
16	6A1	804.55	*	*	*
17	6B11	140.36	*	*	strong

18	6E1	708.91	strong	strong	inferior
19	6E2	-3.76	*	*	*
20	6G7	59.74	strong	strong	inferior
21	7C8	-7.34	*	*	*
22	7G7	-7.27	*	*	*
23	7H9	-6.73	*	*	*
24	8A1	-3.92	*	*	*
25	8A3	784.42	moderate	moderate	*
26	8B4	426.34	*	*	*
27	8C9	-0.59	*	*	*
28	8F6	3.39	*	*	*
29	8F7	-5.56	*	*	*
30	8G8	-7.75	*	*	*
31	8H8	-8	*	*	*
32	10C8	3061.41	*	*	*
33	10D11	-4.86	*	*	*
34	10E7	26.53	strong	strong	moderate
35	11A1	-8.2	*	*	*
36	11B4	-3.76	*	*	*
37	11D12	27.75	moderate	strong	*
38	11H2	-6.2	*	*	*
39	11H7	2.11	strong	strong	inferior
40	12B8	65.83	inferior	strong	*
41	12D5	-6.12	*	*	*
42	13D10	26.14	moderate	moderate	inferior
43	13F12	15.05	*	*	*
44	14A12	-4.66	*	*	*
45	14D4	1.09	moderate	strong	inferior
46	14F12	299.95	strong	strong	*
47	15G11	7.4	moderate	moderate	inferior

Table S3. Hybridoma titer and isotype characterization.

Anti-heroin titers were determined by ELISA against H_{dAc}-BSA. General antibody titers were determined by coating plates with goat anti-mouse Ig antibody and using goat anti-mouse HRP secondary. Isotyping was performed using the same method, but with secondary antibodies that detected the listed heavy and light chain classes.

No.	Hybridoma	Subclone	H_{dAc}-BSA		G a M	
			Titer	Isotype	Titer	Isotype
1	2C10	uncloned	16-32	κ,γ2b	16-32	κ,γ1,γ2b,γ3
2	2D9	uncloned	256+	κ,γ2b	128-256	κ,γ2a,γ2b,γ3,μ
		:1 (G8)	64-128	κ,γ2b	64	κ,γ2b
		:2 (F10)	64	κ,γ2b	32-64	κ,γ2b
		:2 (H7)	64	κ,γ2b	32-64	κ,γ2b
		:2 (C10)	64	κ,γ2b	64-128	κ,γ2b
3	3E12	uncloned	4	γ2a	256+	κ,λ,γ2a,γ2b
4	4G12	uncloned	256+	κ,γ1	256+	κ,γ1,γ2b
		:1 (G4)	256-512	κ,γ1	256	κ,γ1
		:2 (E7)	256-512	κ,γ1	128-256	κ,γ1
		:2 (H5)	512	κ,γ1	256	κ,γ1
5	6B11	uncloned	256+	κ,γ1	256+	κ,γ1,γ3
		:1 (E10)	512	κ,γ1	256	κ,γ1
		:2 (B4)	256	κ,γ1	64-128	κ,γ1
6	6E1	uncloned	256+	κ,γ2b	256+	κ,γ2b,γ3,μ
		:1 (E2)	256	κ,γ2b	256	κ,γ2b
		:2 (C4)	256	κ,γ2b	128-256	κ,γ2b
7	6G7	uncloned	256+	κ,γ2a	256+	κ,γ1,γ2a
8	10D7	uncloned	256+	κ,γ2b,γ3	256+	κ,γ1,γ2b,γ3
9	10E7	uncloned	256+	κ,γ1	256+	κ,γ1,γ3
10	11D12	uncloned	8	λ,γ2a	256+	κ,λ,γ1,γ2a,γ2b
		:1 (H4)	256	λ,γ2a	256	λ,γ2a
		:2 (B4)	256	λ,γ2a	128	λ,γ2a
11	11H7	uncloned	256+	κ,γ1,γ2a	256+	κ,γ1,γ2b,γ3
12	12B8	uncloned	256+	λ,γ2a	256+	λ,γ2a
13	14A7	uncloned	128-256	κ,γ1	256+	κ,γ1,γ2a,γ2b
		:1 (G1)	512-1024	κ,γ1	512-1024	κ,γ1,γ2b
14	14D4	uncloned	64-128	κ,γ1	128	κ,γ1,γ2a
15	14E7	uncloned	32	κ,γ2a	256+	κ,γ1,γ2a,γ2b
16	14F12	uncloned	256+	κ,γ2a	256+	κ,γ2a,μ
17	14H11	uncloned	256+	κ,γ1	256+	κ,γ1,γ2a

Table S4. Statistical analysis of heroin-induced symptoms.

To measure the effect of monoclonal antibody treatment on heroin-induced effects, the appearance of overdose symptoms (death or seizures) was assigned a value of incidence (present = 1, absent = 0) for each mouse regardless of time of symptom onset. Group data was analyzed via the χ^2 distribution. The theoretical frequencies for each mAb group for seizures and death were based on the drug only control group.

Symptom	Group	Symptom Subtype[a]	No. of Mice	χ^2 test goodness-of-fit
Death	4G12	Alive	5	$\chi^2 = 2.386$, $p = 0.06$
		Dead	5	
	11D12	Alive	5	$\chi^2 = 2.386$, $p = 0.06$
		Dead	5	
Seizure[a]	4G12	1	0	$\chi^2 = 30.89$, $p < 0.0001$
		2	18	
		3	18	
		4	0	
	11D12	1	0	$\chi^2 = 1.250$, $p = 0.13$
		2	0	
		3	3	
		4	2	

[a] seizure scores were based on the following scale: 1 = head nodding/twitches, 2 = forelimb clonus/writhing/loss of posture, 3 = running bouncing clonus, 4 = tonic hindlimb extension, 5 = time of death.

Figure S1. Optimization of drug dose for rescue antinociception assay.

Naïve mice (n = 4/group) were injected with different doses of heroin HCl to observe potentials for full antinociception. Mice were tested every 15 minutes until they returned to baseline values.

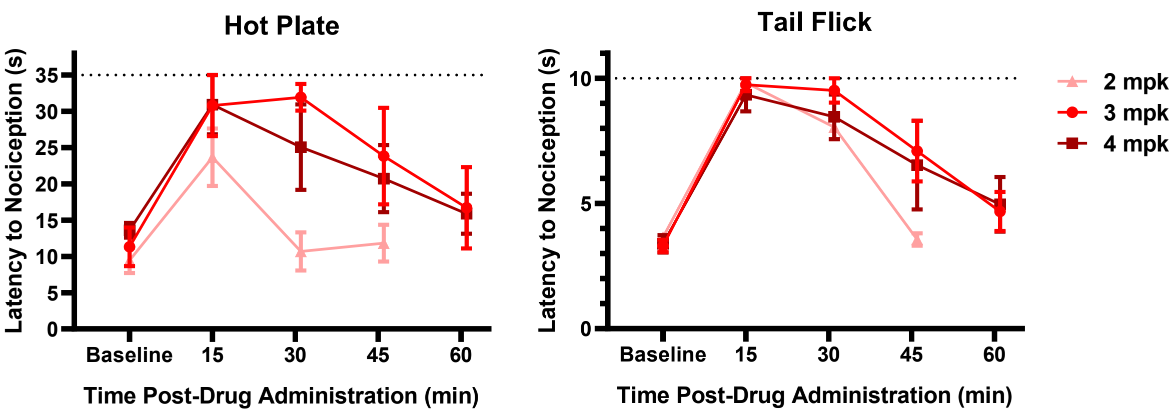

Abbreviations: min, minutes; mpk, milligrams per kilogram; s, seconds.

Figure S2. Quantification of drug and monoclonal antibody concentrations at various timepoints for pharmacokinetic analyses.

Naïve mice (n = 3-4 per time point) were injected with drug, mAb, or drug and mAb and sampled retro-orbitally at various time-points. Drug concentrations were interpolated using the standard curve.

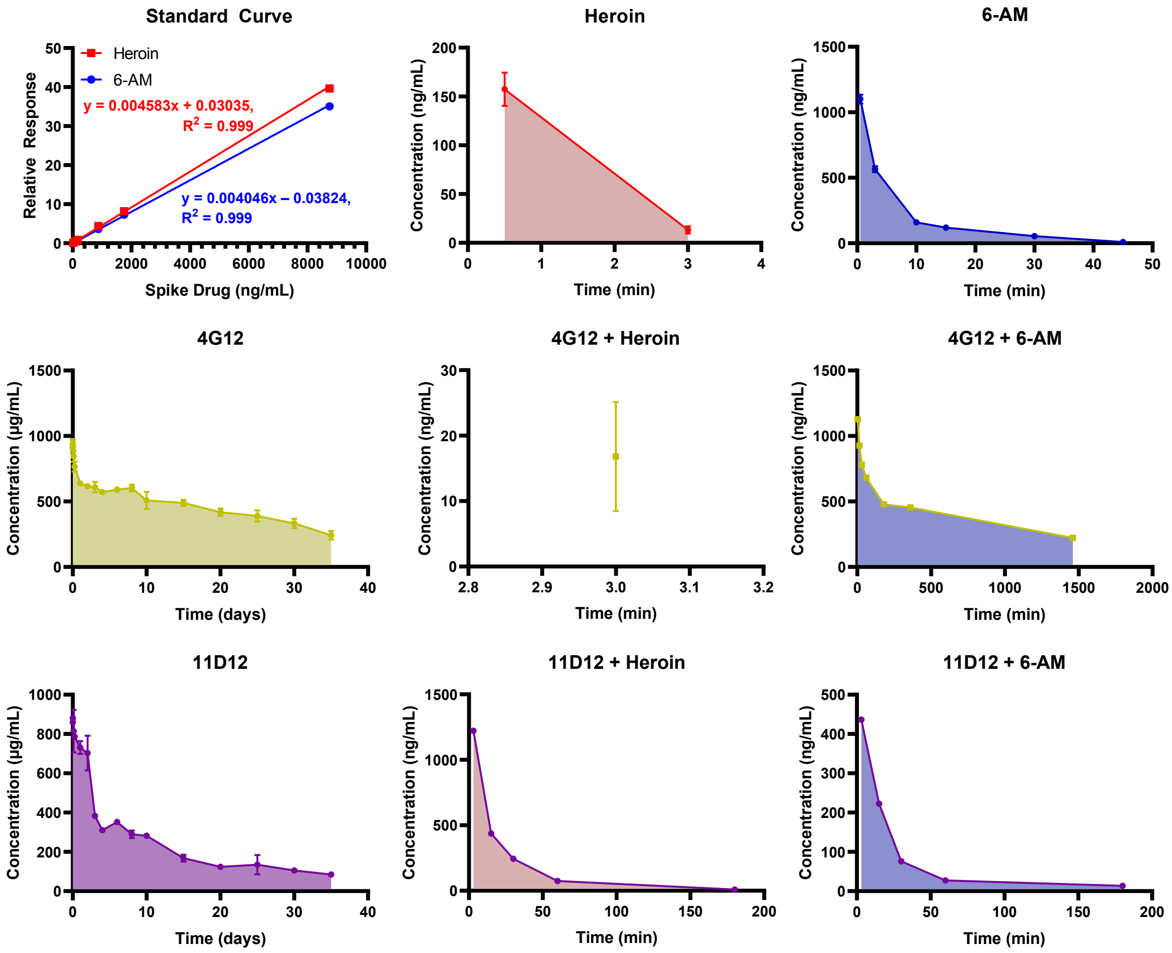

Abbreviations: min, minutes.

References

(1) Belz, T. F.; Bremer, P. T.; Zhou, B.; Ellis, B.; Eubanks, L. M.; Janda, K. D. Enhancement of a heroin vaccine through hapten deuteration. *J. Am. Chem. Soc.* **2020**, *142* (31), 13294-13298. DOI: 10.1021/jacs.0c05219.
(2) Smith, L. C.; Bremer, P. T.; Hwang, C. S.; Zhou, B.; Ellis, B.; Hixon, M. S.; Janda, K. D. Monoclonal Antibodies for Combating Synthetic Opioid Intoxication. *J. Am. Chem. Soc.* **2019**, *141* (26), 10489-10503. DOI: 10.1021/jacs.9b04872 PubMed.
(3) Bremer, P. T.; Schlosburg, J. E.; Lively, J. M.; Janda, K. D. Injection route and TLR9 agonist addition significantly impact heroin vaccine efficacy. *Mol. Pharm.* **2014**, *11* (3), 1075-1080. DOI: 10.1021/mp400631w PubMed.
(4) Jones, J. M.; Raleigh, M. D.; Pentel, P. R.; Harmon, T. M.; Keyler, D. E.; Remmel, R. P.; Birnbaum, A. K. Stability of heroin, 6-monoacetylmorphine, and morphine in biological samples and validation of an LC–MS assay for delayed analyses of pharmacokinetic samples in rats. *J. Pharm. Biomed.* **2013**, *74*, 291-297. DOI: 10.1016/j.jpba.2012.10.033.
(5) Andersen, J. M.; Ripel, Å.; Boix, F.; Normann, P. T.; Mørland, J. Increased Locomotor Activity Induced by Heroin in Mice: Pharmacokinetic Demonstration of Heroin Acting as a Prodrug for the Mediator 6-Monoacetylmorphine in Vivo. *J. Pharmacol. Exp. Ther.* **2009**, *331* (1), 153-161. DOI: 10.1124/jpet.109.152462.
(6) Karinen, R.; Andersen, J. M.; Ripel, Å.; Hasvold, I.; Hopen, A. B.; Mørland, J.; Christophersen, A. S. Determination of Heroin and Its Main Metabolites in Small Sample Volumes of Whole Blood and Brain Tissue by Reversed-Phase Liquid Chromatography-Tandem Mass Spectrometry. *J. Anal. Toxicol.* **2009**, *33* (7), 345-350. DOI: 10.1093/jat/33.7.345 (acccessed 8/10/2022).
(7) Ahmed, T. A. Pharmacokinetics of drugs following IV bolus, IV infusion, and oral administration. *Basic Pharmacokinetic Concepts and Some Clinical Applications* **2015**, *10*.
(8) Urso, R.; Blardi, P.; Giorgi, G. A short introduction to pharmacokinetics. *Eur. Rev. Med. Pharmacol. Sci.* **2002**, *6*, 33-44.
(9) Gabrielsson, J.; Weiner, D. Pharmacokinetic and pharmacodynamic data analysis: concepts and applications. **2001**.
